# Imaging of a Mucocele-Like Lesion of the Breast: A Case Report and Literature Review

**DOI:** 10.7759/cureus.103287

**Published:** 2026-02-09

**Authors:** Pratik Gongloor, Shnutez Doddipalli, James T Roberts, Quan D Nguyen

**Affiliations:** 1 Department of Radiology, University of Texas Medical Branch, Galveston, USA; 2 Department of Radiology, Baylor College of Medicine, Houston, USA

**Keywords:** breast imaging, breast mucocele, invasive mucinous carcinoma, mucocele-like lesion, multilocular mucinous lesion

## Abstract

Mucocele-like lesions (MLLs) of the breast are rare mucin-producing lesions that can closely mimic malignant mucinous neoplasms on imaging and histopathology. We present the case of a 61-year-old woman with a non-palpable MLL of the left breast detected on screening mammography. Mammography demonstrated a 10 mm asymmetry visible on a single mediolateral oblique view in the upper outer quadrant of the left breast, without associated microcalcifications. Targeted ultrasonography did not demonstrate a definitive correlate corresponding to the mammographic finding. Stereotactic core needle biopsy revealed abundant extracellular mucin without malignant cells, raising a differential diagnosis that included a benign MLL versus invasive mucinous carcinoma. Surgical excision confirmed a benign MLL with acellular mucin and focal atypical ductal hyperplasia in adjacent tissue. This case highlights the imaging variability of MLLs and underscores the importance of surgical excision for definitive diagnosis and exclusion of concurrent malignancy.

## Introduction

Mucocele-like lesions (MLLs) of the breast are extremely uncommon mucin-producing lesions of the breast, accounting for approximately 0.4-0.6% of core biopsy specimens [1,2]. They are named due to their resemblance to salivary gland mucoceles, sharing features of ductal mucin accumulation and rupture. MLLs of the breast are characterized by dilated ducts or cysts with extravasation of acellular mucin into the surrounding stroma [3]. Although benign by definition, MLLs are challenging to diagnose because their imaging and histopathologic features can overlap with malignant mucinous neoplasms [4]. MLLs are frequently detected on screening mammography due to associated microcalcifications, which prompt biopsy and guide initial diagnostic consideration [5].

In addition, MLLs become more complex due to their documented association with atypical ductal hyperplasia (ADH), ductal carcinoma in situ (DCIS), and, less commonly, invasive carcinoma. These pathologies may be missed on limited core biopsy sampling due to low cellularity and abundant extracellular mucin [6]. For this reason, surgical excision is generally recommended following a diagnosis of MLL on needle biopsy [7]. In this report, we describe a case of a multiloculated MLL of the breast presenting without mammographic microcalcifications, highlighting an atypical presentation that underscores the variability of MLLs and reinforces the importance of histopathologic confirmation.

## Case presentation

A 61-year-old woman with no history of breast disease presented for routine breast cancer screening. She reported no palpable breast masses on self-examination or clinical examination and denied nipple discharge, skin changes, or breast pain. Her family history was significant for a sister diagnosed with breast cancer in her 50s.

**Figure 1:**
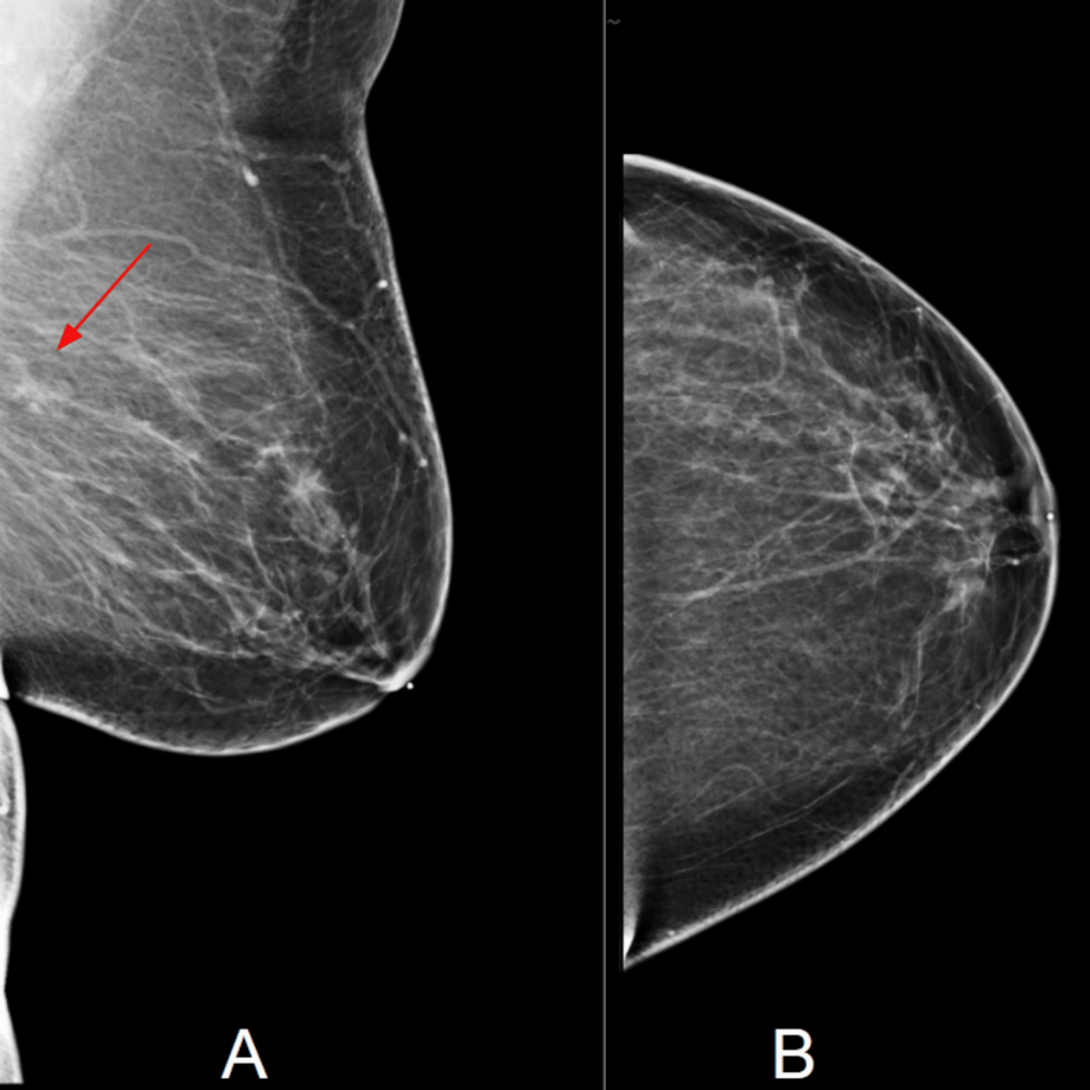
Screening mammograms of the left breast. (A) Mediolateral oblique view, which shows a subtle 10 mm asymmetry (arrow); this was not visible on (B) craniocaudal view.

Screening mammography demonstrated a 10 mm asymmetry in the upper outer quadrant of the left breast on a single mediolateral oblique view. It was located at the 2 o’clock position, posterior depth, approximately 12 cm from the nipple (Figure 1). Additional mammographic views to reduce artifacts, including spot compression or digital breast tomosynthesis, were not performed at the time of the original examination, representing a limitation of this case.

**Figure 2:**
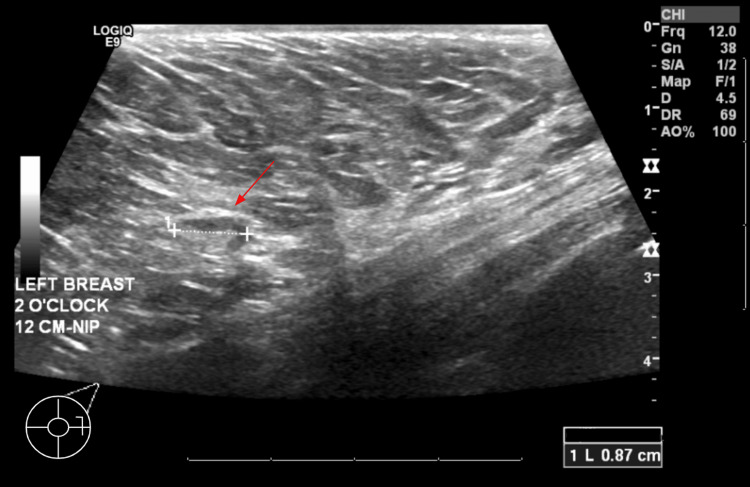
Targeted breast ultrasound. Initial ultrasound of the left breast at the 2 o'clock position, 12 cm from the nipple, demonstrates an intramammary lymph node measuring approximately 8.7 mm.

Targeted breast ultrasound of the region demonstrated a small, oval, circumscribed hypoechoic structure with a central echogenic hilum, consistent with an intramammary lymph node (Figure 2). No sonographic correlate corresponding to the 10 mm asymmetry was identified.

These findings were assessed as Breast Imaging Reporting and Data System (BI-RADS) category 4A, reflecting low suspicion for malignancy given the presence of an asymmetry without associated microcalcifications or architectural distortion. The absence of a reliable ultrasound correlate did not increase the level of imaging suspicion but precluded ultrasound-guided tissue sampling. Therefore, stereotactic-guided core needle biopsy (CNB) was completed using a nine-gauge vacuum-assisted biopsy device (EVIVA), and six core specimens were obtained. Histopathologic evaluation demonstrated a multilocular mucinous lesion, with differential diagnoses that included an MLL and invasive mucinous carcinoma. 

The patient subsequently underwent lumpectomy of the left breast. Surgical pathology confirmed an MLL with acellular mucin, with focal ADH identified in adjacent tissue. Acellular mucin was present at the inked anterior surgical margin, while the inked medial margin was free of ADH with a clearance of 3 mm. Additional background findings included fibrocystic changes with duct ectasia, stromal fibrosis, usual ductal hyperplasia, and microcalcifications. Histological images were unavailable for inclusion due to archiving limitations.

The patient was offered tamoxifen therapy due to a lifetime risk of breast cancer of 19.5% versus an age/race-matched normal population 5.1%, but she declined. The patient recovered well, with mammograms one and three years post-excision demonstrating stable postsurgical changes with no evidence of malignancy, and were assessed BI-RADS category 2 (benign).

## Discussion

MLLs of the breast represent a well-described yet diagnostically challenging entity due to their rarity and overlapping radiologic and histopathologic features with malignant mucinous lesions. Recent studies have proposed that benign MLLs may also harbor or evolve into malignant mucinous neoplasms [8]. They are increasingly recognized as part of a spectrum of mucinous breast pathology consisting of mucin-filled ducts (MFD), MFD with typical or ADH, DCIS, and invasive carcinoma [8,9]. Although MLLs are benign lesions, associated atypical or malignant foci may be undersampled and therefore missed when following current diagnostic recommendations. This case highlights several features that reinforce the need for cautious interpretation and proactive management of mucinous lesions identified on CNB.

MLLs are most commonly detected mammographically due to calcifications, reported in up to 68-93% of cases, often prompting CNB during routine screening [1,10-12]. Ultrasound findings are nonspecific and may demonstrate clustered cysts, complex cystic masses, or heterogeneous hypoechoic lesions, features that substantially overlap with mucinous carcinoma [13]. Our case is unusual in that the lesion lacked calcifications on imaging. Notably, microcalcifications were identified on surgical pathology despite their absence on mammography. This imaging phenotype may reflect low volume or fine microcalcifications below mammographic resolution, or a distribution confined to cystic or stromal compartments. This atypical presentation broadens the recognized imaging spectrum of MLLs and highlights that reliance on calcifications alone may delay detection or lower suspicion in certain cases.

The presence of focal ADH in the adjacent tissue is also clinically significant. Multiple studies have found substantially higher upgrade rates for MLLs when atypia is present (20-50%), compared with less than 3-5% in MLLs without atypia [6,10]. Authors have suggested that clinical follow-up may be recommended as an alternative to surgery in cases with a core biopsy diagnosis of MLL without atypia and discordant imaging findings [6]. In our case, although no atypia or carcinoma were identified on excision, the lack of imaging calcifications and detection of surrounding ADH in tissues may validate the decision for surgical excision as aggressive management of MLLs identified on core biopsy.

MLLs pose inherent challenges for CNB interpretation due to their low cellularity and abundant acellular mucin, particularly when cyst rupture has occurred; a benign CNB result cannot definitively exclude the presence of an adjacent or unsampled malignant component [14,15]. Fine-needle aspiration is even less reliable, with documented cases of MLLs misdiagnosed as mucinous carcinoma due to cytologic overlap [15].

Histopathologic evaluation remains the gold standard for diagnosis. Excision of MLLs is critical as it helps rule out malignancy and provides information for long-term risk assessment. In this patient, the presence of ADH and family history contributed to a recalculated Gail Model lifetime breast cancer risk of 19.5%, substantially higher than the age-/race-matched population risk of 5.1%. Chemoprevention with tamoxifen was offered in accordance with guidelines, though the patient elected surveillance.

## Conclusions

MLLs of the breast are rare benign entities that pose a diagnostic challenge due to their imaging and histopathologic overlap with malignant mucinous neoplasms. This case highlights an atypical presentation of an MLL without mammographic microcalcifications, emphasizing that the absence of calcifications does not exclude the diagnosis. The presence of abundant acellular mucin on CNB, particularly in the setting of low cellularity, underscores the risk of sampling error and the importance of surgical excision for definitive diagnosis. Identification of adjacent ADH further supports excision and informs individualized breast cancer risk assessment and management. Awareness of the variable imaging spectrum of MLLs is essential to ensure appropriate workup, timely excision, and optimal patient counseling.
